# Acute anterior cruciate ligament rupture: can repair become an alternative to reconstruction: a meta-analysis of randomized controlled trials and cohort studies

**DOI:** 10.1186/s13018-024-04812-x

**Published:** 2024-06-02

**Authors:** Michael Opoku, Mingqing Fang, Wenhao Lu, Yusheng Li, Wenfeng Xiao

**Affiliations:** 1 Department of Orthopaedics, Xiangya Hospital, Central South University, 87 Xiangya Road, Changsha, Hunan 410008 China; 2 Xiangya School of Medicine, Central South University, Changsha, Hunan 410083 China; 3 National Clinical Research Center for Geriatric Disorders, Xiangya Hospital, Central South University, Changsha, Hunan 410008 China

**Keywords:** Anterior cruciate ligament, Repair, Reconstruction, Meta-analysis, Rupture

## Abstract

**Purpose:**

To perform a meta-analysis to compare clinical outcomes of anterior cruciate ligament (ACL) repair and ACL reconstruction for acute ACL rupture.

**Method:**

We searched Pubmed, Embase, the Cochrane Library, and Web of Science databases to seek relevant studies. Clinical outcomes included failure rate, hardware removal rate, anteroposterior (AP) knee laxity, and patient-reported outcomes. In addition, subgroup analysis was carried out according to repair techniques, rupture locations, and study designs. Funnel plots were used to detect publication bias. All statistical analysis was performed using STATA (version 14.2, StataCorp).

**Results:**

A total of 10 articles were included in this study, comprising 5 randomized controlled trials (RCTs) and 5 cohort studies, involving a total of 549 patients. We found no statistical differences between the ACL repair and ACL reconstruction in the following outcomes: failure rate, AP knee laxity, International Knee Documentation Committee (IKDC) score, Lysholm score, Knee Injury and Osteoarthritis Outcome (KOOS) Score, and Tegner score. However, the ACL repair group had a higher hardware removal rate. Except for AP knee laxity results on different repair techniques, there was no statistical difference in other subgroup analyses.

**Conclusion:**

Compared with ACL reconstruction, ACL repair shows similar results in clinical outcomes, and it is promising to be an effective alternative treatment for acute ACL rupture. Larger samples and higher-quality studies are needed to support our results and further explore the advantages of ACL repair in other aspects.

**Level of evidence:**

Level III.

**Supplementary Information:**

The online version contains supplementary material available at 10.1186/s13018-024-04812-x.

## Introduction

ACL tear is a very common ligament injury, with an annual incidence of about 68.6 cases per 100,000 people, accounting for more than 50% of knee injuries [1], leading to chronic knee instability and seriously affecting athletic capabilities and overall quality of life of patients. Female athletes are more likely to suffer anterior cruciate ligament (ACL) rupture than male athletes [2] and also have a higher risk of medial meniscus tear [3]. The first open ACL repair was reported in 1895 and was widely used until the 1980s [4]. Although short-term outcomes of surgery are encouraging, medium- and long-term outcomes reported high rates of instability, reinjury, and poor functional scores [5]. Finally, at the end of the last century, surgeons abandoned ACL repair and moved firmly toward ACL reconstruction because of its outstanding clinical outcomes [6], such as restoring knee function and returning to preinjury activity levels [7].

ACL reconstruction has been the gold standard for the treatment of ACL injuries since then. However, it is still not completely satisfactory because of its high rates of failure and some complications [8, 9]. Even in 2020, ACL reconstruction in high-risk populations was associated with graft failure rates of 18–28% and reoperation rates of approximately 7–15% [10]. The use of autologous grafts has a certain risk of causing pain and weakness at the donor site [11], and may also lead to a permanent loss of bone mineral content [12], while the use of allografts carries a risk of disease transmission. Fleming JD et al. reported in a meta-analysis that proprioception improved to some extent after ACL reconstruction, but could not return to the normal level [13]. Considering that repair techniques may address some of these issues, and the recent advances in imaging diagnosis, arthroscopy, and rehabilitation techniques, ACL repair has gained renewed attention [14]. Effective rehabilitation is important and necessary to achieve full and successful recovery, restore knee function, and return to sports after an ACL surgery [6, 15–17].

Many different repair techniques have emerged in the last decade [18]. These techniques vary, including SAR (suture anchor repair) with suturing of the ACL with fixation to the femoral footprint with anchors directly, IBLA (Internal brace ligament augmentation) with an internal brace added to the initial fixation for increased strength, DIS (dynamic intraligamentary stabilization) with implantation of a dynamic screwspring mechanism in the tibia and repair of the ACL, and BEAR (bridge-enhanced ACL repair) with a biological scaffold [19–23].

Several studies have shown that proximal ACL tears have a healing capacity akin to that of the medial collateral ligament, enabling the attainment of acceptable clinical outcomes to be reported in ACL repair for Sherman type I and II ACL injuries [24–26]. Furthermore, promising clinical studies to have also been published, showcasing the efficacy of repair techniques in addressing mid-substance tears [27–30]. However, a prevailing debate persists regarding the viability of repair techniques as a potential alternative, or even as a superior approach, to ACL reconstruction [31, 32]. Existing meta-analyses reveal that ACL repair is associated with higher failure rates, increased hardware removal rates, and amplified knee joint laxity compared to reconstruction [33, 34]. It is crucial to note, however, that the majority of the included studies are characterized by a lower tier of quality and misconceptions. And another limitation of previously published meta-analysis is the inclusion of papers reporting results of old open techniques and studies with a non-selected population when indication for repair was not based on of tear type and injury to surgery intervals. Defining the terms “acute” and “chronic” in anterior cruciate ligament (ACL) rupture is very crucial in the decision-making process and treatment plan. Flint et al. [35] defined acute and chronic ACL ruptures in a systematic review as ≤ 6 weeks and ≥ 6 months respectively. Van der List et al. reported that early repair was more likely to successfully repair a torn ACL than delayed repair [36]. Jorjani et al. repaired acute ACL rupture in patients with time from injury to surgery less than 6 weeks and achieved good clinical outcomes in the medium to long term [37]. In light of this, we performed a meta-analysis incorporating a more rigorous selection of RCTs and cohort studies, aiming to conduct a more nuanced comparison of the impacts of repair and reconstruction in the management of primary *acute ACL injuries*, thereby enabling the derivation of more robust conclusions.

Therefore, the purpose of this meta-analysis is to critically evaluate and compare the failure rate, hardware removal rate, AP knee laxity, and patient-reported outcomes between repair and reconstruction, and further explore whether there are differences between different repair techniques, rupture locations, and study designs.

## Methods

The PRISMA (Preferred Reporting Items for Systematic Reviews and Meta-Analyses) guidelines were used to design our meta-analysis [38]. The protocol and considerations of this systematic review and meta-analysis were registered in the International Prospective Register of Systematic Reviews (PROSPERO) on November 11, 2023, ID: CRD42023475078.

### Literature search

Two authors independently conducted searches in the following electronic databases: PubMed, the Cochrane Library, Embase, and Web of Science, targeting articles available from inception until 22nd March 2024. The search strategies employed a combination of entry words and Medical Subject Headings (MeSH) terms, focusing on key terms or phrases: (“Anterior Cruciate Ligament”) AND (“ repair” OR “reinsertion” OR “reattachment” OR “healing” OR “suture” OR “dynamic intrafilamentary stabilization” OR “internal brace” OR “bridge-enhanced”) AND (“ replacement “OR “reconstruction”), with no limitation to language. To augment the comprehensiveness of our search, supplementary searches were undertaken by examining articles included in systematic reviews as well as reference lists in other relevant articles, to identify studies not initially retrieved from the databases.

### Inclusion and exclusion

Firstly, the retrieved literature was de-duplicated. Subsequently, two authors independently screened the titles and abstracts of the remaining articles, adhering strictly to predefined inclusion and exclusion criteria. Concurrently, a manual screening process was executed on the references retrieved from systematic reviews to identify relevant studies that satisfied the established criteria. The full text of each piece of qualifying literature was then acquired to ascertain whether it warranted inclusion. Discrepancies in literature selection were primarily resolved through deliberation between the two authors aiming for a consensus. If disagreements persisted, the decision was deferred to a third author The inclusion and exclusion criteria of this meta-analysis were as follows. Inclusion criteria: (1) RCTs or Cohort studies; (2) studies comparing primary ACL repair with reconstruction; (3) any of the subsequent clinical outcomes were reported (failure rate, hardware removal rate, AP knee laxity (ΔATT assessed by calculating the mean difference obtained by subtracting the value from the uninjured knee from that of the injured knee), IKDC score [39], KOOS score [40], Lysholm score [41], and Tegner score [42].); (4) a minimum of 1-year follow-up; (5) studies where the time from injury to surgery was within six weeks in the repair group (≤ 6 weeks); (6) arthroscopy was used. Exclusion criteria: (1) non-clinical studies such as in vitro or animal studies; (2) studies where the full text of the literature was not available; (3) patients involving revision surgery or previous knee injuries.

### Data extraction

Two authors independently undertook the data extraction process from the finalized included literature, utilizing a structured literature information table. In instances of discrepancies, initial efforts were aimed at resolution through negotiation to attain consensus; failing this, the decision was escalated to a third author. The extracted data included the following aspects: (1) basic characteristics of the literature: title, first author, and year of publication; (2) experimental information: study design, level of evidence, surgical methodologies employed within the repair and reconstruction groups; (3) Patient information: number of patients in each group, gender ratio, age, duration of follow-up, location of ligament rupture, and time from injury to operation; (4) outcomes as previously mentioned.

### Quality assessment

The authors utilized the Cochrane Collaboration’s risk of bias tool [43] and NOS (Newcastle-Ottawa Scale) [44] to assess the risk of bias in RCTs and cohort studies, respectively. Disagreements were initially aimed to be resolved through negotiation between the two authors (XL AND GY); failing that, the decision was deferred to a third author (BZJ). The Cochrane scale incorporates 7 items: (1) random sequence generation; (2) allocation concealment; (3) blinding of participants and personnel; (4) blinding of outcome assessment; (5) Incomplete outcome data; (6) selective reporting; (7) Other bias. Each item was categorized as low risk, high risk, or unclear. NOS scale comprises 3 domains: (1) selection (subdivided into 4 parts, each section scoring up to one point); (2) comparability (a subsection, scoring a maximum of two points); (3) outcome (segmented into two parts, each part will get a maximum of one point). Each study could attain a maximum of nine points, with scores interpreted as follows: 7–9 (good), 5–7 (fair), 3–5 (relatively fair), and 0–2 (poor).

### Statistical analysis

All data analyses were conducted using the meta package in STATA (version 14.2, StataCorp). For continuous variables, such as patient-reported outcomes and ΔATT, data were synthesized using means and standard deviations, or medians and quartiles. The inverse variance method was applied for pooling, and results were presented as weighted mean differences (WMD) with a 95% confidence interval (CI). For dichotomous variables, such as failure rate and hardware rate, essential data, including the number of events and total patient counts, were extracted. Subsequently, the Mantel-Haenszel (MH) method was utilized for data synthesis. Pooled effect sizes were presented in the form of risk ratios (RR) with a 95% CI. An effect size with a P value less than 0.05 was considered statistically significant. Heterogeneity between studies was assessed using the I^2^ statistic. According to Cochrane’s handbook, a fixed-effect model was applied when the I^2^ value was less than 50%; otherwise, a random-effect model was deemed appropriate. Subgroup analyses were performed based on repair techniques, rupture locations, and study designs, considering P values less than 0.05 as indicative of significant differences between subgroups [45]. Funnel plots were used to detect publication bias [46].

## Results

### Literature selection

**Fig. 1:**
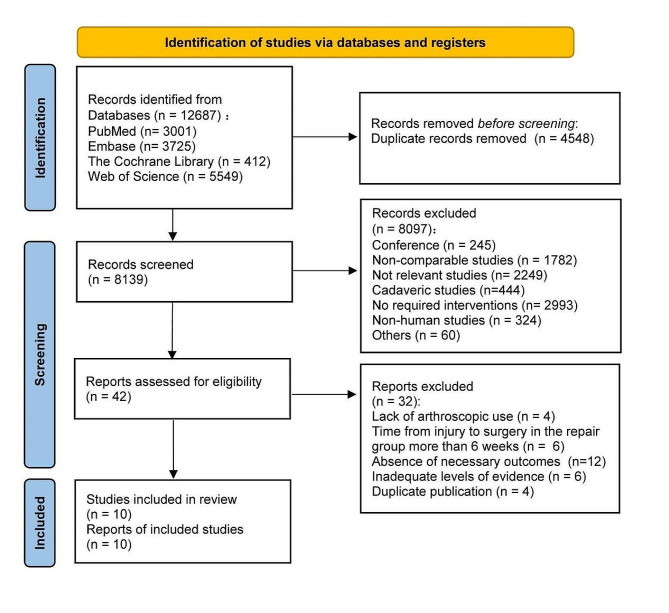
Flow chart of literature search and screening

A comprehensive search yielded a total of 12,687 articles from all databases. Following the removal of 4,548 duplicate entries, 8,139 articles were screened based on specific inclusion and exclusion criteria. Upon reviewing titles and abstracts, 42 articles were shortlisted for further evaluation. After a detailed full-text review, 32 articles were subsequently excluded due to various reasons such as lack of arthroscopic use (*n* = 4), absence of necessary outcomes (*n* = 12), inadequate levels of evidence (*n* = 6), non-acute ACL repair (*n* = 6), and duplicate publication (*n* = 4). Ultimately, 10 articles [28, 47–55], consisting of 5 RCTs [47–50, 52] and 5 cohort studies [28, 51, 53–55], were deemed eligible for inclusion in this study, with 2 articles [47, 50] emanating from a singular study. Figure 1 shows the flow chart of systematic literature search and screening.

### Basic characteristics of the literature

The 10 articles [28, 47–55] included involved 9 trials containing 549 patients. The studies were published in English-language journals between 2018 and 2023. The sample size was 20 to 100 patients, with a mean age of 17 to 39.5 years. Patients were followed for a mean time of 12 to 60 months. The proportion of male patients was 40–74%, and the mean time from injury to surgery was 13 to 36 days in the ACL repair group. Among them, 3 articles [47, 50, 51] published on 2 RCTs included patients with central ACL rupture, while the remaining articles primarily focused on patients with proximal ACL rupture. For the classification of repair techniques, two studies used SAR [53, 55] and one used IBLA [54], BEAR was employed in 3 articles [47, 50, 51], and DIS was utilized in 4 articles [28, 48, 49, 52]. We subsequently conducted subgroup analyses of outcomes according to the repair techniques, rupture locations, and study designs. The basic characteristics of the included studies are shown in Table 1.

**Table 1:** Characteristics of the included studies

First author-Year	Study Design-LOE	Rupture location		Intervention		Follow-up (years)	Sex(Male /Female)		Age(Years)		Time from injury to surgery(Days)		Sample size	
		Rp	Rs	Rp	Rs		Rp	Rs	Rp	Rs	Rp	Rs	Rp	Rs
Yang-2022	PCT-II	proximal	proximal	SAR	semimembranosus and semitendinosus tendon	1	11/5	11/8	37.0 ± 9.66	39.53 ± 12.81	16.5 ± 6.63	16.5 ± 6.63	16	19
Murray-2019	PCT-II	Length of tibial remnant, % 50–74: 90% 75–100:10%	Length of tibial remnant, % 50–74: 60% 75–100:40%	BEAR	Semitendinosus-gracilis tendon	2	4/6	2/8	24.1 ± 4.9	24.6 ± 5.5	20.8 ± 4.8	52.9 ± 16.7	10	10
Barnett-2021	RCT-I	Length of tibial remnant, % 50–74: 88% 75–100:12%	Length of tibial remnant, % 50–74: 88% 75–100:12%	BEAR	Semitendinosus-gracilis tendon (*n* = 33), bone–patellar tendon–bone (*n* = 2)	2	28/37	16/19	17 (16–20)	17 (15–23)	36 (29–42)	39 (33–43)	65	35
Schliemann-2017	RCT-I	NA	NA	DIS	Semitendinosus tendon	1	15/15	22/8	28.2 ± 11.4	29.1 ± 12.0	15.2 ± 4.5	16.3 ± 5.0	30	30
Hoogeslag-2022	RCT-I	Proximal:83.3% Central:12.5% Distal:4.2%	NA	DIS	semitendinosus tendon	5	19/5	18/6	21.0 (10.0–27.0)	22.0 (19.3–25.0)	13 (12–16)	47 (42–71)	24	24
Glasbrenner-2022	RCT-I	Proximal:90.7% Midsubstance:9.3%	Proximal:76.2% Midsubstance:23.8%	DIS	semitendinosus tendon	5	25/18	31/11	28.7 ± 11.4	27.6 ± 10.6	14.5 ± 5.2	16.2 ± 7.3	43	42
Murray-2020	RCT-I	Length of tibial remnant, % 50–74: 88% 75–100:12%	Length of tibial remnant, % 50–74: 88% 75–100:12%	BEAR	semitendinosus-gracilis tendon (*n* = 33), bone–patellar tendon–bone (*n* = 2)	2	28/37	16/19	17 (16–20)	17 (15–23)	36 (29–42)	39 (33–43)	65	35
Kayaalp-2022	RCS-III	proximal or middle third	NA	DIS	semitendinosus tendon	4	12/3	24/6	27.8 ± 9.5	27.4 ± 10.2	15.4 ± 14	49.7 ± 19.6	15	30
Muller-2023	RCS-III	proximal	NA	IBLA	semitendinosus or gracilis tendon	2	13/16	13/14	36.8 ± 10.6	37.0 ± 10.7	20 (15–25)	28(14–56)	29	27
Ferretti-2023	PCT-II	proximal	proximal	SAR	hamstring tendon	2	32/21	30/17	32.7 ± 12	25.4 ± 11.1	9.2 ± 2.9	9.1 ± 2.6	53	47

Data are expressed as Mean ± standard deviation or median (interquartile range); Rp, repair; Rs, Reconstruction; LOE, level of evidence; RCT, randomized controlled trial; PCT, Prospective cohort study; RCS Retrospective cohort study; NA, not available; M, male; F, female

### Quality assessment

Among the 10 included articles, a total of 5 were RCTs, and the remaining 5 were cohort studies. The assessment results about the risk of bias have been systematically illustrated in Tables 2 and 3. For the RCT studies, a pervasive risk of bias was noted, with a solitary exception [47, 50] where all evaluative items were rated low. A universal application of allocation concealment was observed across all studies, and a rigorous adherence to random sequence generation was maintained in 4 studies. The main source of risk of bias is the implementation of blinding. For cohort studies, NOS scale scores ranged from 7 to 9, and all were of good quality, but only one of the studies received full marks [55].

**Table 2:** Quality assessment of the RCT studies

Study-Year	selection bias		performance bias	detection bias	attrition bias	reporting bias	Other bias
	Random sequence generation	Allocation concealment	Blinding of participants and personnel	Blinding of outcome assessment	Incomplete outcome data	Selective reporting	
Barnett-2021	low	low	low	low	low	low	low
Schliemann-2017	unclear	low	unclear	unclear	low	low	unclear
Hoogeslag-2022	low	low	unclear	unclear	low	low	unclear
Glasbrenner-2022	low	low	unclear	unclear	high	low	unclear
Murray-2020	low	low	low	low	low	low	low

**Table 3:** Quality assessment of the non-RCT studies

Study	Selection	Comparability	Outcome	Total Score
Murray-2019	****	**	**	8
Yang-2022	****	**	**	8
Kayaalp-2022	***	*	***	7
Muller-2023	****	*	***	8
Ferretti-2023	****	**	***	9

### AP knee laxity

**Fig. 2:**
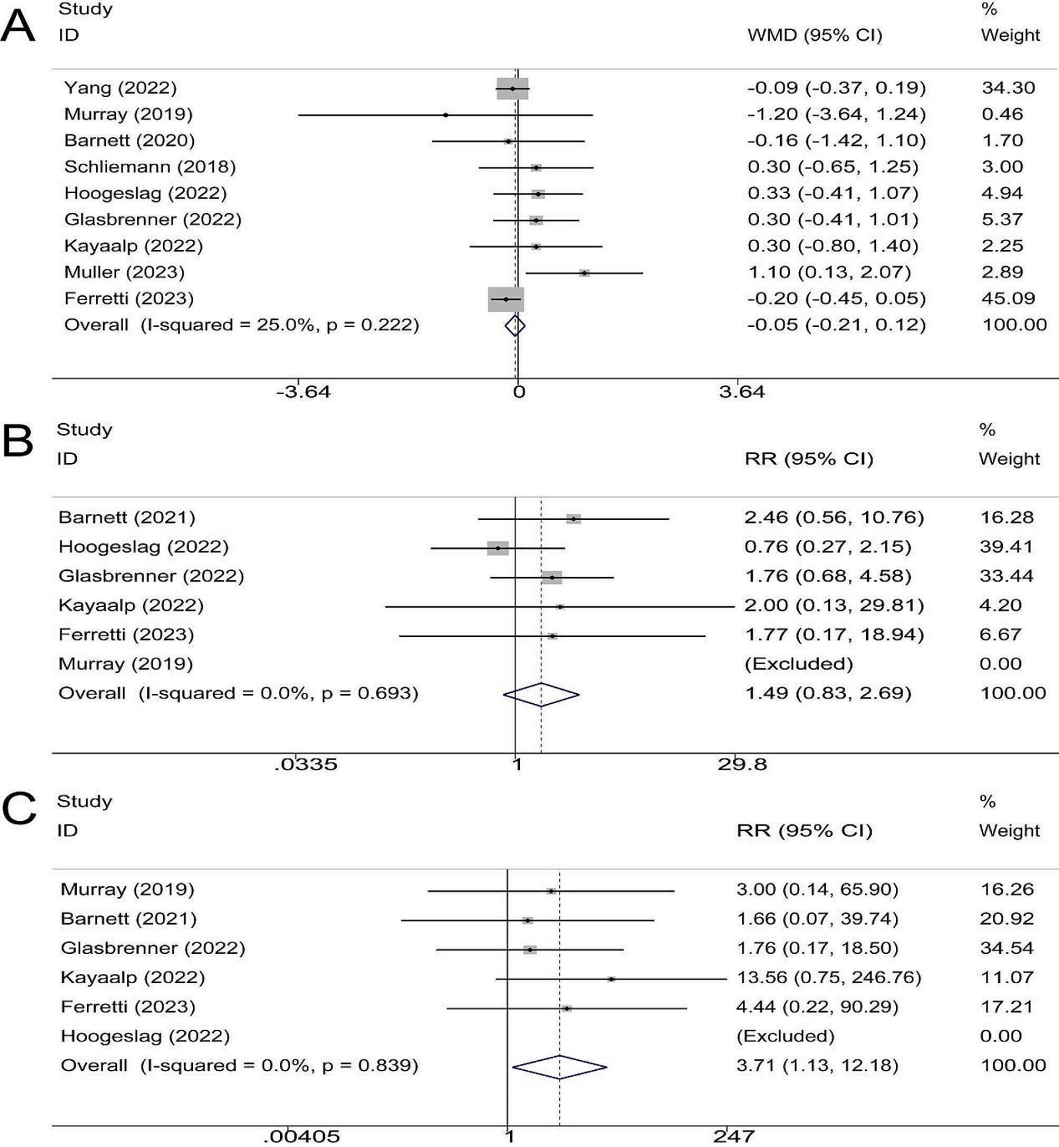
Meta-analysis of (**A**) AP knee laxity; (**B**) Failure rate and (**C**) Hardware removal rate

9 trials comprising 262 and 251 patients in the repair and reconstruction groups, respectively, reported ΔATT; 2 were followed up for 1 year, 4 were followed up for 2 years, and 3 were at least 4 years. The results of the meta-analysis showed that the repair was not inferior to reconstruction in terms of ΔATT (WMD, -0.05; 95%CI, -0.21 ∼ 0.12; *P* = 0.559; I^2^ = 25.0%) (Fig. 2A).

### Failure rate

Failure was defined as ACL rerupture or combination of findings at physical examinations and subjective instability on the injured side. A total of 6 clinical trials comprising 200 in the repair group and 174 patients in the reconstruction group, reported failure rates; 3 trials had a follow-up time of 2 years, and 3 trials had a follow-up time of at least 4 years. The results of the meta-analysis showed that there was no significant difference in the postoperative failure rate between the two groups (RR, 1.49; 95% CI, 0.83 ∼ 2.69; *P* = 0.182; I^2^ = 0.0%) (Fig. 2B).

### Hardware removal rate

Hardware removal rate was reported in 6 trials, comprising 200 in the repair group and 174 patients in the reconstruction group; 3 trials were followed up for 2 years, and 3 trials were at least 4 years. The results of the meta-analysis showed that compared with reconstruction, repair had higher hardware removal rates (RR, 3.71; 95% CI, 1.13 ∼ 12.18; *P* = 0.031; I^2^ = 0.0%) (Fig. 2C).

### IKDC score

**Fig. 3:**
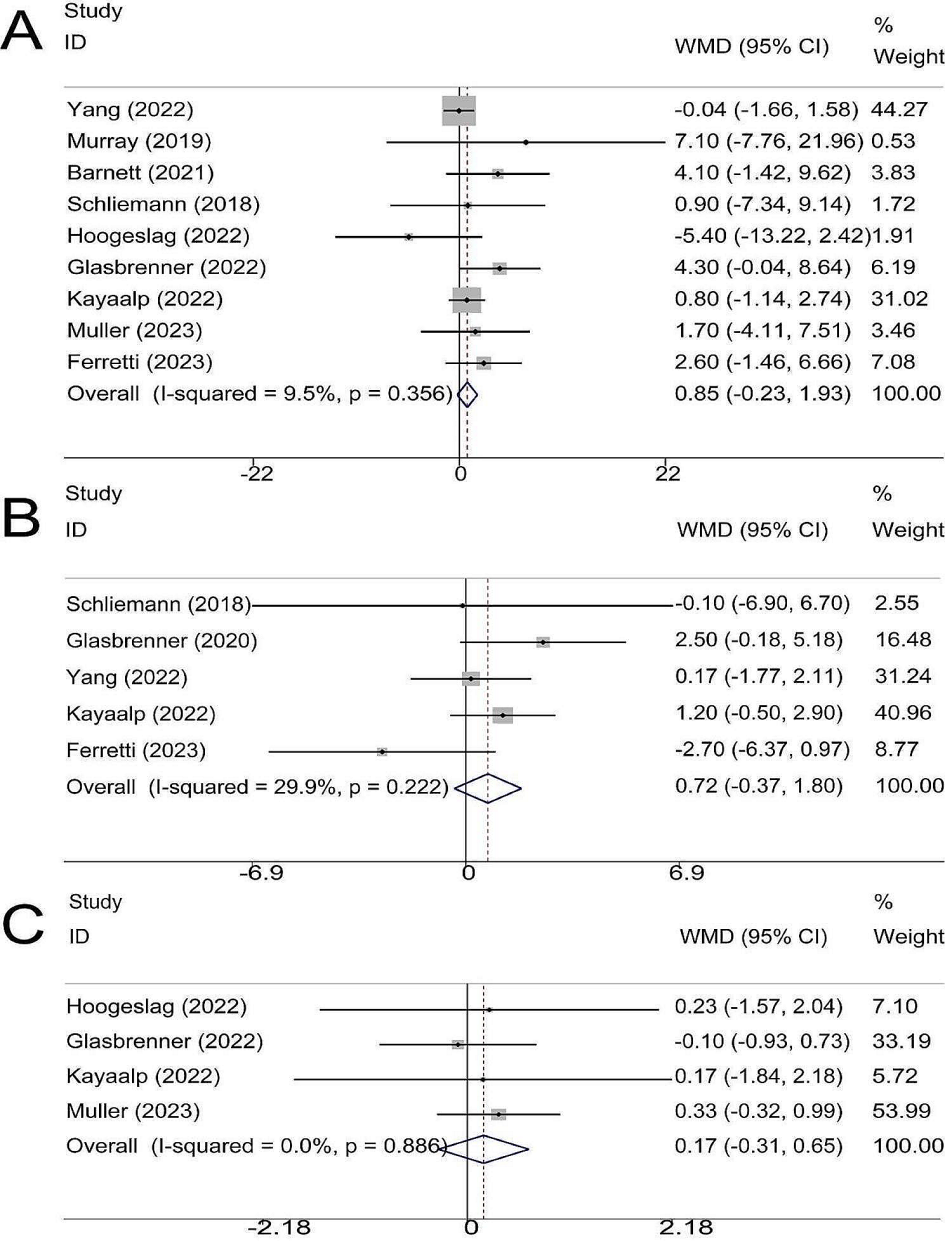
Meta-analysis of (**A**) IKDC score; (**B**) Lysholm score and (**C**) Tegner score

9 trials comprising 269 and 244 patients in the repair and reconstruction groups, respectively, reported IKDC scores; 2 studies had a follow-up time of 1 year, 4 trials had a follow-up time of 2 years, and 3 trials had a follow-up time of at least 4 years. The results of the meta-analysis showed that repair was as good as reconstruction in IKDC score (WMD, 0.85; 95% CI, -0.23 ∼ 1.93; *P* = 0.125; I^2^ = 9.5%) (Fig. 3A).

### Lysholm score

Lysholm score was reported in 5 trials, comprising 146 in the repair group and 155 patients in the reconstruction groups; 4 trials were with follow-up of 2 years, and 1 was at least 4 years. The results of the meta-analysis showed that there was no significant difference in Lysholm scores between the two groups (WMD, 0.72; 95% CI, -0.37 ∼ 1.80; *P* = 0.196; I^2^ = 29.9%) (Fig. 3B).

### Tegner score

Tegner score was only reported in 4 clinical trials with a follow-up of 2 ∼ 5 years. The results of the meta-analysis showed that compared with reconstruction, repair had a similar result (WMD, 0.17; 95% CI, -0.31 ∼ 0.65; *P* = 0.481; I^2^ = 0.0%) (Fig. 3C).

### KOOS

**Fig. 4:**
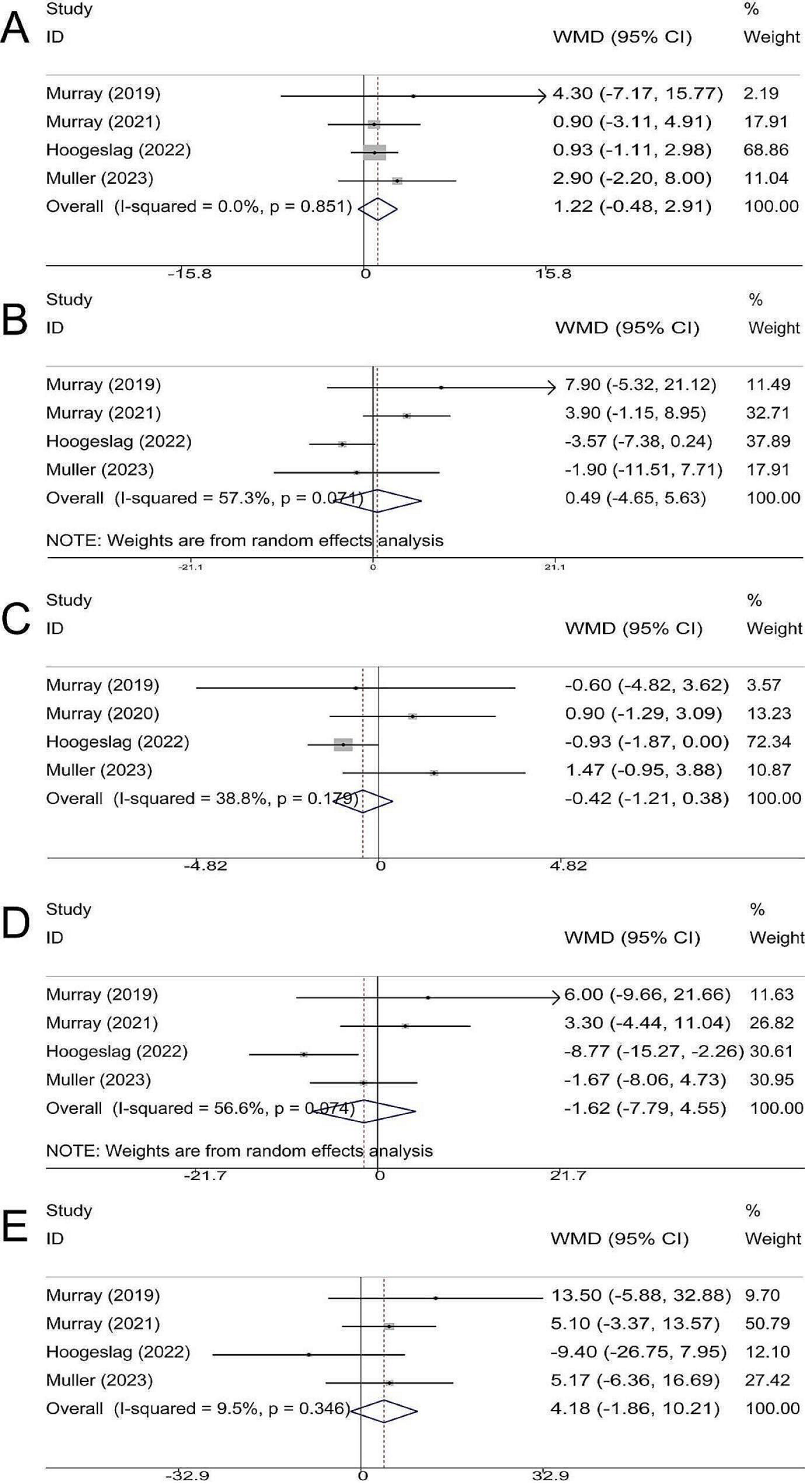
Meta-analysis of (**A**) KOOS-Pain; (**B**) KOOS-Symptoms; (**C**) KOOS-ADL; (**D**) KOOS-SR and (**E**) KOOS-QoL

KOOS was reported in 4 studies; the duration of follow-up was 2 years in 2 trials, and at least 4 years in 2 trials. There were no statistical differences in Pain (WMD, 1.22; 95% CI, -0.48 ∼ 2.91; *P* = 0.159; I^2^ = 0.0%) (Fig. 4A), Symptoms (WMD, 0.49; 95% CI, -4.65 ?∼ 5.63; *P* = 0.851; I^2^ = 57.3%) (Fig. 4B), ADL (Activities of daily living) (WMD, -0.42; 95% CI, -1.21 ∼ 0.38; *P* = 0.304; I^2^ = 38.8%) (Fig. 4C), SR (Sport and Recreation) (WMD, -1.62; 95% CI, -7.79 ∼ 4.55; *P* = 0.608; I^2^ = 56.6%) (Fig. 4D) and QoL (Quality of life) (WMD, 4.18; 95% CI, -1.86 ∼ 10.21; *P* = 0.175; I^2^ = 9.5%) (Fig. 4E).

Due to the limited number of studies, we only performed subgroup analysis for two outcomes (AP knee laxity and IKDC score). The subgroup analysis included three aspects: repair techniques, ligament rupture location, and study designs. The results of the summarized subgroup analysis are shown in Table 4. In addition to the significant differences in ΔATT between different surgical methods (*P* = 0.021), the p values of other subgroup analyses were all greater than 0.05, indicating that there was no statistical difference between subgroups.

**Table 4:** Subgroup analysis of AP knee laxity AND IKDC score

Subgroups	No. of studies	AP knee laxity				IKDC score			
		WMD (95% CI)	I^2^	*P* value	*P* for subgroups	WMD (95% CI)	I^2^	*P* value	*P* for subgroups
Study design									
non-RCT	5	-0.10 (-0.28, 0.08)	48.8%	0.256	0.319	0.59 (-0.57, 1.75)	0.0%	0.318	0.245
RCT	4	0.26 (-0.17,0.68)	0.0%	0.233		2.46 (-0.47,5.38)	40.5%	0.099	
Main rupture location									
proximal	7	-0.04 (-0.21, 0.12)	38.6%	0.622	0.559	0.68 (-0.42, 1.79)	11.0%	0.227	0.161
midstance	2	-0.38 (-1.50,0.74)	0.0%	0.507		4.46 (-0.71,9.64)	0.0%	0.091	
Repair technique									
SAR	2	-0.15 (-0.34, 0.03)	0.0%	0.106	0.021	0.32 (-1.18, 1.83)	28.6%	0.674	0.480
IBLA	1	1.10 (0.13, 2.07)	-	0.026		1.70 (-4.11, 7.51)	-	0.566	
BEAR	2	-0.38 (-1.50,0.74)	0.0%	0.507		4.46 (-0.71,9.64)	0.0%	0.091	
DIS	4	0.31 (-0.11, 0.73)	0.0%	0.145		1.04 (-0.65, 2.74)	37.9%	0.226	

### Publication bias

**Fig. 5:**
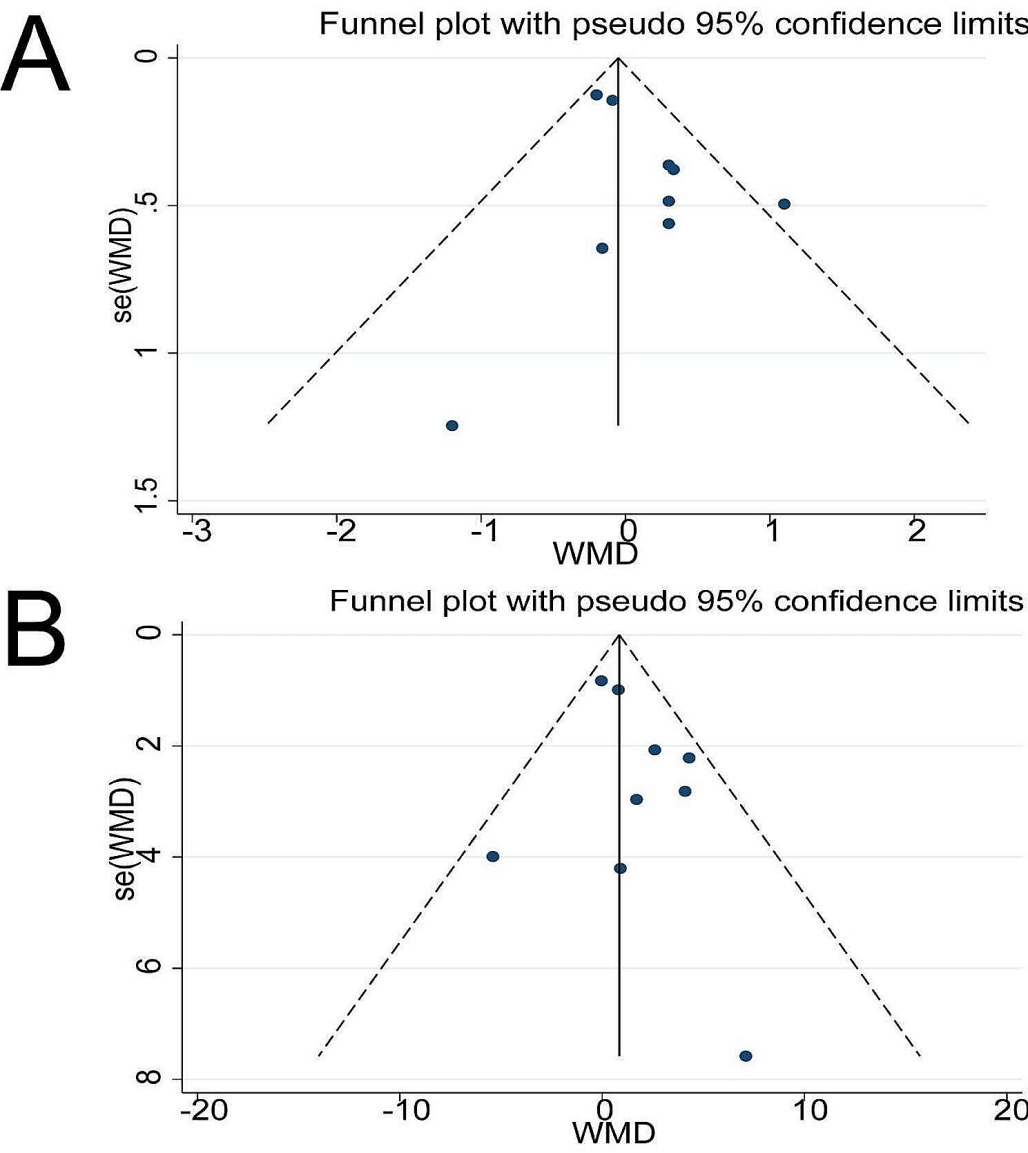
Funnel plot of meta-analysis (**A**) AP knee laxity; (**B**) IKDC score

We detected publication bias for only two outcomes, AP knee laxity and IKDC score, due to the limited included studies. As shown in Fig. 5, the funnel plots showed no obvious visual asymmetry, so no significant publication bias was detected.

## Discussion

This meta-analysis aimed to evaluate and compare the clinical outcomes of ACL repair and reconstruction in acute ACL rupture based on RCTs and cohort studies. The significant finding of this meta-analysis was that the repair techniques had similar clinical results compared with the reconstruction in terms of failure rate, AP knee laxity, IKDC score, KOOS score, Lysholm score, IKDC score, and Tegner score, but with higher hardware removal rates. The higher hardware removal rate in the repair group compared to the reconstruction group in this study may be attributed to the fact that high number of patients who had a DIS technique. Due to the need to withstand very high tensile load, the monobloc spring- screw used in the DIS surgery group was bulkier than that used in ACL reconstruction, which may have contributed to the high hardware removal rates reported in the previous literature [56, 57]. Upon conducting subgroup analysis, considering different repair techniques, rupture locations, and study designs, there were no remarkable differences observed except the AP knee laxity results of different repair techniques. Hence, acute ACL repair demonstrates considerable potential as an alternative treatment to ACL reconstruction.

There are four phases of histology in the time-dependent histological response to ACL rupture, namely an inflammatory phase, an epiligamentous repair phase, a proliferative phase, and a remodeling phase. During the epiligamentous repair phase, synovial tissue forms, covering the end of the ruptured ACL [58]. Most of the synovial lining cells were myofibroblast-like cells, which may be partly responsible for the retraction of the ruptured ACL and thus may impede repair of the torn ACL. Repairing the ACL within 6 weeks, before depilatory cells form and the ligament retracts, can promote ligament healing, which may be why repair can achieve similar results to reconstruction [59].

With the proliferation of literature on ACL repair, many systematic reviews exploring repair techniques have been published. These reviews predominantly summarize and critically evaluate the prevailing status of repair techniques and their associated clinical outcomes: treatment outcomes for proximal ACL ruptures are deemed acceptable, showing no significant disparities across various repair techniques [10, 60, 61]. However, two meta-analyses [33, 62] comparing ACL repair with reconstruction have unveiled some disconcerting results. Pang et al. [62] compared repair with reconstruction, reporting increased knee joint laxity (WMD, 0.56; 95% CI, 0.04–1.08). Contrastingly, our meta-analysis did not demonstrate any statistically significant differences in AP knee laxity (WMD, -0.05; 95%CI, -0.21 ∼ 0.12) after follow-up. A notably narrow confidence interval characterized our findings on knee laxity, implying enhanced precision and reliability, supported further by the incorporation of RCTs and cohort studies, conducive to generating more accurate outcomes. Migliorini et al. [33] reported a higher failure rate (OR 2.63; 95% CI 1.36–5.08) in the repair group compared to the reconstruction group, but one study [63] included in their analysis was an RCT conducted in the last century, which exhibited a relatively high failure rate in the repair group (OR 13.46; 95% CI 1.76–103.25). Given the rudimentary level of repair technology available at the time, the study may be biased. In contrast, our results indicate that although the failure rate in the repair group was marginally higher compared to the reconstruction group, the difference was not statistically significant (RR, 1.49; 95% CI, 0.83 ∼ 2.69; *P* = 0.182).

Since the end of the 20th century, ACL reconstruction has become the gold standard surgical treatment in surgical intervention for ACL rupture. A historical review of ACL surgeries suggests that the initial desuetude of ACL repairs was primarily due to immature arthroscopic techniques and imprecise patient selection, culminating in suboptimal outcomes post-open ACL repairs [64]. Consequently, Li et al. [64] discerned notable differences between open and arthroscopic repairs, concluding that arthroscopic approaches garnered superior clinical outcomes. In 1991, Sherman et al. reported the important finding that patients with proximal tears with good tissue quality tended to have significantly better clinical outcomes than patients with other types of tears [65]. After this revelation, the preponderance of clinical investigations pivoting on repair techniques has predominantly centered around proximal anterior cruciate ligament tears, yielding satisfactory outcomes. Recent reviews approved this perspective, highlighting the efficacy of repair techniques in proximal tears with good to excellent tissue quality. This aligns seamlessly with the conclusions drawn from our study. Intriguingly, our meta-analysis encompassed two trials with three studies [47, 50, 51], wherein the BEAR technique was predominantly employed on patients exhibiting mid-substance ACL ruptures, and with excellent clinical outcomes. This observation potentiates a reevaluation of the applicability of specialized repair methodologies like BEAR, broadening the horizons for repair technique indications. However, a circumspect interpretation is warranted, given that the trials emanated from a singular team and are not substantiated by extensive longitudinal follow-ups.

Frobell et al. compared early and delayed reconstruction and found that although the two methods had comparable functional scores, the delayed group had higher rates of meniscectomy and medial ventricular osteoarthritis [66, 67]. Vermeijden et al. [68] conducted a retrospective comparative cohort study that compared the outcomes of early and delayed repairs, ascertaining that both acute and delayed primary ACL repairs yield analogous clinical and functional outcomes in short to mid-term follow-up. Contrary to prevalent assumptions that ACL repair is only applicable to acute rupture, these findings instigate a reevaluation of prior perceptions, albeit necessitating further research for robust substantiation. Additionally, Vermeijden et al. [69]reported that younger patients were more likely to have surgery failure than older patients (37% vs. 3.5%), and Ferreiraden et al. [70] showed similar results. This finding is consistent across studies, suggesting that a comprehensive evaluation of the effect of age on ACL repair is essential to clarify indications for repair applications.

Theoretically, arthroscopic ACL repair has the advantage of maintaining proprioception over ACL reconstruction. In Yang et al.‘s study [53], repair was not superior to reconstruction in proprioception at the last follow-up time, as both techniques eventually facilitated a return to normal proprioceptive function; however, proprioception was recovered earlier in the repair group. Furthermore, since the repair technique does not require graft removal, it holds theoretical promise for better muscle strength recovery. Barnett et al. [47] reported better Hamstring muscle strength at a 2-year follow-up, while Kayaalp et al. [28] noted a higher ACL-RTS with ACL repair at 6 months, indicating improved psychological readiness in patients to return to sports. These key findings, albeit not included in this meta-analysis due to limited studies, illustrate that compared to reconstruction, repair exhibits certain advantages. More research is needed to explore whether there are significant benefits of repair techniques in proprioception, muscle strength, and psychological readiness.

The meta-analysis performed by us has the following advantages: (1) In the process of making this meta-analysis, a rigorous and systematic search, and supplementation of literature were employed to make the evidence we synthesized more credible; (2) RCTS and cohort studies were included, and the baseline of the original literature was comparable; (3) Multiple outcomes were included in this meta-analysis to comprehensively compare reconstruction and repair from various aspects; (4) We included studies where the time from injury to surgery was within six weeks (≤ 6 weeks) in the repair group, and studies where arthroscopy was used. This may provide more accurate and reliable results since primary repair tends to be more successful in the acute phase.

Certainly, this systematic review also has some shortcomings. There were only a small number of long-term follow-up literature. In terms of outcomes, this meta-analysis did not compare the differences between reconstruction and repair in non-key outcomes, such as proprioception, muscle strength, and mental readiness.

## Conclusion

Compared with ACL reconstruction, emerging primary repair of acute ACL tear techniques have similar results in terms of failure rate, AP knee laxity, IKDC score, KOOS score, Lysholm score, and Tegner score. On the basis of the reported results, injury to surgery interval seems to be an important factor in ACL repair surgery and should be considered as an indication for this technique. This study believes that ACL repair can replace reconstruction as an appropriate surgical method to a certain extent, but the indication and careful selection of patients are crucial to be considered. Larger samples and higher-quality studies are needed to support our results and further explore the advantages of multiple surgical approaches such as primary repair, augmentation, and reconstruction in other aspects.

### Electronic supplementary material

Below is the link to the electronic supplementary material.

Supplementary Material 1

## Data Availability

No datasets were generated or analysed during the current study.

## Declarations

### Registration

The study protocol was registered on the PROSPERO website (CRD42023475078).

## Abbreviations

ACL: Anterior Cruciate Ligament
SAR: Suture Anchor Repair
IBLA: Internal Brace Ligament Augmentation
DIS: Dynamic Intraligamentary Stabilization
BEAR: Bridge-Enhanced ACL Repair
AP: Anteroposterior
PRISMA: Preferred Reporting Items for Systematic Reviews and Meta-Analyses
IKDC: International Knee Documentation Committee
NOS: Newcastle-Ottawa Scale
WMD: Weighted Mean Difference
CI: Confidence Interval
RR: Risk Ratio
RCTs: Randomized Controlled Trials
PCS: Prospective Cohort Study
RCS: Retrospective Cohort Study
ΔATT: The Mean Difference in Anterior Tibial Translation between the injured and Contralateral Knees
KOOS: Knee Injury and Osteoarthritis Outcome Score
ADL: Activities of Daily Living
SR: Sport and Recreation
QoL: Quality of Life
